# Draft Genome Sequence of an Isolate of *Fusarium oxysporum* f. sp. *melongenae*, the Causal Agent of *Fusarium* Wilt of Eggplant

**DOI:** 10.1128/genomeA.01597-16

**Published:** 2017-02-16

**Authors:** Zhangyong Dong, Tom Hsiang, Mei Luo, Meimei Xiang

**Affiliations:** a Department of Plant Pathology, Zhongkai University of Agriculture and Engneering, Guangzhou, Guangdong, China; b School of Environmental Sciences, University of Guelph, Guelph, Ontario, Canada

## Abstract

Here, we present the genome sequence of an isolate (14004) of *Fusarium oxysporum* f. sp. *melongenae*, an eggplant pathogen. The final assembly consists of 1,631 scaffolds with 53,986,354 bp (G+C content, 46.4%) and 16,485 predicted genes.

## GENOME ANNOUNCEMENT

*Fusarium* species are some of the most important phytopathogenic and toxigenic fungi, causing various diseases on nearly every economically important plant species. Aside from their economic importance, species of *Fusarium* also serve as key model organisms for biological research (1).

*Fusarium* wilt of eggplant caused by *Fusarium oxysporum* f. sp. *melongenae* is an economically important soilborne disease limiting eggplant production worldwide. This pathogen was initially reported in Japan in 1958 (2), and the first report in China was in 2005 (3). Here, we report a draft assembly for *F. oxysporum* f. sp. *melongenae* isolate 14004, a field strain originally collected in Guangdong Province, China, where *Fusarium* wilt of eggplant was observed over 10 years ago and is currently endemic in the area.

The mycelium of fungal hyphae was grown on cellophane over potato dextrose agar (PDA), and 500 mg of mycelium was used for DNA extraction with the Qiagen DNeasy plant minikit (Qiagen, Mississauga, Canada); then, 1 μg of genomic DNA was sent for sequencing at Génome Québec (Montreal, Canada), specifying 100-bp paired-end reads with a 300-bp insert. Over 43 million paired-end reads totaling 10.7 Gb were received. The genome was assembled using the programs Velvet version 1.2.10 (4), ABySS version 2.0.1 (5), and SOAP*denovo* version 2.04 (6), with odd-numbered kmers between 21 and 91. Assembly quality was assessed by examining the *N*_50_ value and by examining the total number of scaffolds produced by the programs. The initial assembly was 54,488,475 bp in length, with an *N*_50_ value of 568,281 bp, resulting in 4,617 scaffolds. After removing scaffolds that were smaller than 200 bp by using a perl script, the final assembly consisted of 1,631 scaffolds with a genome size of 53,986,354 bp (G+C content, 46.4%). A total of 16,485 protein-coding genes were predicted from the assembly, with the highest *N*_50_ (kmer = 77, ABySS) using AUGUSTUS version 3.2.2 (7) based on gene models from *Fusarium oxysporum*.

### Accession number(s).

This whole-genome shotgun project has been deposited at DDBJ/ENA/GenBank under the accession no. MPIL00000000. The version described in this paper is version MPIL01000000.
